# The inclusion of canola meal or corn-DDGS in reduced-crude protein corn-soybean meal diets modified digestibility, microbial metabolites, and cecal microbiota independent of coccidiosis vaccination regime in broiler chickens

**DOI:** 10.1016/j.psj.2025.105763

**Published:** 2025-09-01

**Authors:** June Hyeok Yoon, Adeleye M. Ajao, Shravani Veluri, Revathi Shanmugasundaram, Adelumola Oladeinde, Jeferson Lourenco, Oluyinka A. Olukosi

**Affiliations:** a Department of Poultry Science, University of Georgia, Athens, GA 30602, USA; b Toxicology and Mycotoxin Research Unit, USDA-ARS, Athens, GA 30605, USA; c Egg and Poultry Production Safety Research Unit, USDA-ARS, Athens, GA 30605, USA; d Department of Animal and Dairy Science, University of Georgia, Athens, GA 30602, USA

**Keywords:** Coccidiosis vaccination, Reduced-protein diet, Canola meal, Corn DDGS, Soybean meal

## Abstract

The present study aimed to evaluate the effect of incorporating canola meal (**CM**) and corn distiller’s dried grains with solubles (**cDDGS**) into corn-soybean meal (**SBM**)-based reduced-crude protein (**RP**) diets on growth performance, nutrient digestibility, and cecal short-chain fatty acids (**SCFA**) and microbiota in broiler chickens with or without coccidiosis vaccination. Upon arrival, birds were assigned to treatments in 2 × 4 (vaccination × diet) factorial arrangement, with half of birds spray-vaccinated against coccidiosis. Birds were allocated to vaccinated or unvaccinated groups in a randomized complete block design, then divided into 4 dietary treatments with 6 replicates (27 birds/pen) in a split-plot design on d 7. Birds received a common starter diet from d 0-7. Birds were fed one of four dietary treatments during grower (day 7-28) and finisher (day 28-42) phases: (1) corn-SBM-based standard protein diet (**SP-SBM**), (2) RP corn-SBM-based diet (**RP-SBM**), (3) RP-SBM in which SBM was replaced with 80 g/kg CM (**RP-CM**), and (4) RP-SBM in which SBM was replaced with 100 g/kg cDDGS (**RP-cDDGS**). The RP diets had 40 or 30 g/kg less crude protein than SP diet in each phase. No vaccination × diet interactions were observed, except for jejunal gene expression of *occludin* (*P* = 0.029). Vaccination reduced (*P* = 0.041) body weight gain (**BWG**) on d 7. Birds fed SP diet tended (*P* = 0.064) to have higher BWG and body weight on d 42 than RP diets. Apparent ileal digestibility of most AA was lower (*P* < 0.05) in birds fed RP-CM and cDDGS diets compared to the SP diet. The RP-cDDGS group had the highest (*P* < 0.05) cecal branched-chain fatty acid concentrations on d 42. *Pseudobutyricicoccus* enriched in birds fed RP-cDDGS diet was positively correlated (*P* < 0.05) with cecal isovalerate concentrations. In conclusion, coccidiosis vaccination had no effect on responses during grower and finisher phases. Replacing SBM with CM or cDDGS in RP diets may compromise nutrient digestibility and lead to unfavorable cecal metabolite profiles.

## Introduction

Coccidiosis, an intestinal disease in animals caused by *Eimeria* protozoa, is one of the most prevalent diseases in poultry production. The financial burden and economic losses associated with coccidiosis are primarily due to impaired growth performance, reduced nutrient utilization, and immune system activation in infected birds. In recent decades, ionophore and chemical coccidiostats have been widely used as feed additives to protect against coccidiosis in commercial poultry production. These compounds effectively prevent *Eimeria* infections by reducing oocyst shedding, thereby improving growth performance and minimizing lesion scores (Behnamifar et al., 2019; Rogala-Hnatowska et al., 2024). However, the use of ionophore coccidiostat has raised concerns regarding antibiotic resistance, as global surveillance has identified increasing levels of multidrug-resistant bacterial pathogens in humans (Parker et al., 2021). Consequently, vaccination against coccidiosis has emerged as a viable alternative for controlling *Eimeria* infections in poultry production (Gaghan et al., 2022). Recent advancements in new-generation vaccines aim to enhance commercial viability by broadening coverage against prevalent *Eimeria* species without inducing resistance in field strains, as observed with anticoccidial ionophores (Zaheer et al., 2022). Commercially available gel bead and spray *Eimeria* vaccines have demonstrated comparable protective effects on growth performance and gut lesion scores; however, spray vaccines may facilitate more effective vaccine oocyst cycling in the litter, enhancing flock immunity (Albanese et al., 2018; Jenkins et al., 2013).

Despite the potential benefits, previous studies have reported inconsistent effects of coccidiosis vaccination on broiler performance. For instance, Lee et al. (2011) found that vaccinated broilers exhibited reduced growth performance compared to unvaccinated birds, particularly during the early stages (d 13-17); however, this reduction was no longer evident by the end of the study (d 27). This reduction during the early stage may be attributed to the fact that coccidiosis vaccines contain live, sporulated oocysts in varying mixtures and concentrations of *Eimeria* species (Albanese et al., 2018), which initiate an immunologic response in birds and prioritize immune development over growth. In contrast, Gautier et al. (2019) reported no significant differences in growth performance between vaccinated and unvaccinated broilers up to 36 days of age.

The reduced-crude protein (**RP**) diets have been extensively studied for their potential to mitigate environmental impacts of nonruminant animal production. By lowering dietary nitrogen (**N**) concentration, RP diets not only reduce feed costs but also improve gut health and litter quality by minimizing N emissions in excreta (Strifler et al., 2023). To date, most research on RP diets have used corn and soybean meal (**SBM**) as the primary protein sources (Cho et al., 2024; Olukosi et al., 2024). The SBM is well known for its highly digestible amino acid (**AA**) composition. However, alternative protein sources, such as canola meal (**CM**) and corn distillers’ dried grains with solubles (**cDDGS**), also provide comparatively balanced digestible AA profiles and high digestibility (Stein et al., 2016). On the other hand, CM and cDDGS contain approximately twice the concentration of insoluble dietary fiber (**IDF**) compared with SBM (Acosta et al., 2020; Navarro et al., 2018), which may impair nutrient digestibility and alter intestinal metabolites in the gastrointestinal tract due to a shortened digesta retention time (Zhang et al., 2023). Such differences could, in turn, influence the growth performance of broilers. In line with this supposition, our previous study reported that partial replacement of SBM with CM or cDDGS negatively affected immune responses in RP corn-SBM diets for *Eimeria*-challenged broilers (Shanmugasundaram et al., 2024). Therefore, using the same replacement level of CM and cDDGS as in our previous study, the present study aimed to address the research gap regarding how different protein sources can be included in RP diets by evaluating growth performance, nutrient utilization, and intestinal metabolites in broiler chickens with or without coccidiosis vaccination.

The present study hypothesized that coccidiosis vaccination would result in growth retardation, particularly in the early life stage. Furthermore, following coccidiosis vaccination, the partial incorporation of CM and cDDGS into RP diets was anticipated to further compromise the performance of vaccinated broilers during subsequent growth stages. Therefore, the objective of the present study was to investigate the effects of partly replacing SBM with CM or cDDGS in corn-SBM-based RP diets. Responses of interest were the treatment outcomes on growth performance, ileal AA digestibility, relative gene expression of nutrient utilization and tight junction proteins, and cecal short-chain fatty acid (**SCFA**) and microbiota composition in broiler chickens following coccidiosis vaccination or not.

## Materials and methods

All animal experiment procedures used in the present study were approved by the Institutional Animal Care and Use Committee at the University of Georgia.

### Coccidiosis vaccination

A total of 1,440 male by-product broiler chicks were obtained from a Cobb hatchery (Cleveland, GA, USA). Upon arrival, half of the birds were transported to the Poultry Diagnostic Research Center at the University of Georgia for coccidiosis vaccination (Coccivac-B52; Merck & Co., Inc., Rahway, NJ, USA). The birds were vaccinated in a spray cabinet in accordance with the manufacturer’s instructions. The vaccine was a live oocysts vaccine containing a mixture of *Eimeria* species, including *E. acervulina, E. maxima, E. maxima MFP, E. mivati, E. tenella*.

### Dietary treatments

All birds received the same starter diet from day 0 to 7, formulated to meet the energy and nutrient recommendations outlined in the Cobb 500 Broiler Performance & Nutrition Supplement (2022). Four experimental diets were formulated for the grower (d 7-28) and finisher (d 28-42) phases (Table 1). The four diets were: (1) standard crude protein diet (**SP**; 200 g/kg CP during the grower phase and 180 g/kg CP during the finisher phase), (2) reduced-CP corn-SBM-based diet (**RP-SBM**; 160 g/kg CP for the grower phase and 150 g/kg CP for the finisher phase), (3) reduced-CP corn-SBM-CM-based diet (**RP-CM**; replacement of approximately 60 g/kg SBM with 80 g/kg CM in the grower phase and 85 g/kg CM in the finisher phase), (4) reduced-CP corn-SBM-cDDGS diet (**RP-cDDGS**; replacement of approximately 40 g/kg of SBM with 100 g/kg cDDGS in the grower phase and 110 g/kg cDDGS in the finisher phase). Experimental diets within each phase were isocaloric across all diets. The RP diets were formulated to contain similar standardized ileal digestible AA concentrations according to Cobb 500 recommendations. Glycine equivalents (**Gly_equi_**) were maintained at 12.0 and 11.3 g/kg for the grower and finisher diets, respectively. Dietary electrolyte balance was kept constant at 246 and 235 mEq/kg for the grower and finisher phases, respectively, to minimize potential adverse effects on the growth performance of broilers. Titanium dioxide (3 g/kg) was included in the starter and grower diets as an indigestible index for determining apparent ileal digestibility (**AID**) of AA. All diets were supplemented with phytase (Quantum Blue, AB Vista, Marlborough, UK) at 0.1 g/kg, providing 500 FTU/kg. One phytase unit (FTU) is defined as the amount of enzyme that releases 1 µmol of inorganic phosphorus per minute from sodium phytate at 37 °C and pH 5.5. The starter diet was fed in crumble form, and the grower and finisher diets were provided as pellets. The analyzed composition of the diets is shown in Table 2.

**Table 1:** Ingredient and nutrient compositions of experimental diets (as-fed basis, g/kg)^1^^,^^2^.

		Grower diets				Finisher diets				
Items	Starter diet	SP	RP-SBM	RP-CM	RP-cDDGS		SP	RP-SBM	RP-CM	RP-cDDGS
Ingredients										
Corn	583.3	637.5	732.3	707.8	680.4		681.2	749.1	718.0	693.2
Soybean meal	356.0	306.0	204.0	146.0	157.0		255.0	181.0	120.0	128.0
Corn distillers’ dried grains with solubles	–	–	–	–	100.0		–	–	–	110.0
Canola meal	–	–	–	80.0	–		–	–	85.0	–
Soybean oil	25.0	25.0	12.0	12.0	9.0		35.0	24.0	28.0	20.0
Dicalcium phosphate	17.5	8.0	8.7	8.0	6.8		6.5	7.0	6.7	5.2
Limestone	5.5	8.2	8.4	7.8	9.8		8.7	8.7	7.7	10.2
L-Lys-HCl	0.7	1.1	4.7	5.3	5.9		1.6	4.2	4.9	5.6
DL-Met	2.0	1.5	2.1	2.0	2.0		1.5	1.9	1.8	1.8
L-Thr	0.3	0.2	1.9	2.0	2.1		0.2	1.4	1.6	1.8
L-Trp	–	–	0.1	0.2	0.2		–	0.3	0.4	0.5
L-Val	–	–	1.2	1.3	1.4		–	1.0	1.1	1.2
L-Arg	–	–	2.6	3.2	3.7		–	2.5	3.1	3.7
L-Cys	–	0.9	1.5	1.5	1.5		0.9	1.5	1.5	1.5
L-Gly	–	–	2.1	2.1	2.1		–	2.1	2.1	2.1
L-Ile	–	–	0.9	1.3	1.3		–	0.8	1.1	1.2
L-Ser	–	–	2.6	2.6	2.6		–	2.6	2.6	2.6
L-Phe	–	–	–	–	–		–	–	0.5	0.2
Titanium dioxide	3.0	3.0	3.0	3.0	3.0		–	–	–	–
NaHCO_3_	2.0	2.0	2.0	2.0	1.5		2.0	2.0	2.0	1.5
NaCl	2.8	3.5	3.5	3.5	3.2		3.5	3.5	3.5	3.2
Vitamin premix^3^	1.0	1.0	1.0	1.0	1.0		1.0	1.0	1.0	1.0
Trace mineral premix^4^	0.8	0.8	0.8	0.8	0.8		0.8	0.8	0.8	0.8
KCO_3_		1.2	4.5	6.5	4.6		2.0	4.5	6.5	4.6
Phytase (Quantum Blue)	0.1	0.1	0.1	0.1	0.1		0.1	0.1	0.1	0.1
Total	1,000	1,000	1,000	1,000	1,000		1,000	1,000	1,000	1,000
Calculated nutrients and energy										
Crude protein	222	201	160	160	161		180	151	151	151
Metabolizable energy, kcal/kg	2,902	2,967	2,992	2,979	2,996		3,080	3,089	3,097	3,091
Total Ca	7.8	6.3	6.3	6.3	6.2		5.9	5.9	5.9	5.9
Total P	6.9	5.1	4.8	5.1	4.8		4.6	4.5	4.8	4.4
Available P^5^	4.3	2.6	2.6	2.5	2.5		2.3	2.2	2.2	2.3
dEB, mEq/kg	248	244	249	247	246		234	238	235	236
Digestible amino acids										
Arg	14.9	13.3	12.5	12.6	12.5		11.6	11.6	11.6	11.6
His	6.0	5.4	4.3	4.2	4.2		4.9	4.1	4.0	3.9
Ile	9.6	8.6	7.5	7.6	7.6		7.6	6.9	6.9	6.9
Leu	19.0	17.7	14.9	14.3	15.4		16.2	14.2	13.6	14.8
Lys	12.7	11.6	11.6	11.6	11.6		10.6	10.6	10.6	10.6
Met	5.4	4.7	4.7	4.7	4.7		4.4	4.4	4.4	4.4
Cys	3.6	4.2	4.3	4.4	4.2		3.9	4.1	4.2	4.1
Phe	10.9	9.9	7.8	7.3	7.6		8.8	7.3	7.3	7.2
Thr	8.7	7.8	7.8	7.9	7.8		7.0	7.0	7.0	7.0
Trp	2.6	2.3	1.8	1.9	1.8		2.0	1.9	1.9	1.9
Val	10.5	9.6	8.8	8.8	8.8		8.6	8.1	8.1	8.1
Met + Cys	9.1	8.9	9.0	9.1	8.9		8.4	8.5	8.6	8.5
Phe + Tyr	19.0	17.3	13.6	12.8	13.3		15.4	12.8	12.4	12.6
Gly	7.2	4.9	5.4	5.7	5.5		4.1	5.0	5.4	5.1
Ser	9.2	8.4	9.2	8.9	9.1		7.5	8.8	8.4	8.7
Gly + Ser	16.4	13.3	14.6	14.6	14.6		11.6	13.8	13.8	13.8
Gly_equi_^6^	13.7	10.9	12.0	12.0	12.0		9.40	11.3	11.4	11.3

1 SP, standard protein diet; RP, reduced protein; SBM, soybean meal; CM, canola meal; cDDGS, corn distillers’ dried grains with solubles; dEB, dietary electrolyte balance; Gly_equi_, glycine equivalents.

2 Experimental phases were divided into three separate stages: starter (day 0 to 7), grower (day 7 to 28), and finisher (day 28 to 42).

3 Vitamin premix supplemented the following per kg of diets: vitamin A, 5,484 IU; vitamin D_3_, 2,643 IU; vitamin E, 11 IU; niacin, 44.1 mg; D-pantothenic acid, 11 mg; riboflavin, 5.49 mg; vitamin B_6_, 2.92 mg; menadione sodium bisulfite, 4.38 mg; thiamin mononitrate 2.2 mg; folic acid 990 mg; biotin, 55.2 mg; vitamin B_12_, 13.2 mg; choline chloride, 771 mg; pyridoxine hydrochloride 3.3 mg.

4 Trace mineral premix supplemented the following per kg of diets: iodine, 1.11 mg; manganese, 66.06 mg; copper, 4.44 mg; iron, 44.1 mg; zinc, 44.1 mg; selenium, 300 mg.

5 The available phosphorus concentration was calculated based on the matrix value for phytase at 500 FTU/kg of feed (Quantum Blue, AB Vista, Marlborough, UK).

6 Gly_equi_ was calculated using the following equation: concentration of Gly + 0.714 × concentration of Ser.

**Table 2:** Analyzed nutrient compositions of the experimental diets (as-fed basis, g/kg)^1^^,^^2^.

		Grower diets				Finisher diets			
	Starter diet	SP	RP-SBM	RP-CM	RP-cDDGS	SP	RP-SBM	RP-CM	RP-cDDGS
Dry matter	865	865	864	866	869	860	866	865	864
Nitrogen	34.7	32.5	28.8	29.1	29.8	27.8	25.1	24.9	25.8
Neutral detergent fiber		80.1	74.0	102.9	101.6	75.3	82.1	92.2	112.9
Acid detergent fiber		31.9	27.7	44.0	36.5	27.6	26.9	38.6	36.6
Indispensable amino acids									
Arg	13.8	13.1	12.4	12.1	13.1	10.6	10.4	10.4	11.4
His	5.6	5.5	4.5	4.3	4.6	4.5	3.8	3.8	4.0
Ile	9.3	9.0	7.9	7.8	8.2	7.4	6.6	6.2	7.0
Leu	17.8	18.3	15.9	14.8	16.8	15.5	13.5	13.1	15
Lys	12.9	12.2	12.3	12.2	12.8	10.3	10.0	10.4	10.8
Met	4.9	4.8	4.5	4.5	5.0	3.7	3.8	4.0	4.1
Phe	10.6	10.3	8.4	7.8	8.4	8.6	7.3	7.3	7.6
Thr	8.3	7.9	7.8	8.1	8.3	6.6	6.6	6.9	7.2
Trp	2.4	2.3	1.8	1.8	1.8	1.9	1.7	1.8	1.7
Val	10.4	10.4	9.4	9.5	9.9	8.5	8.0	8.2	8.4
Dispensable amino acids									
Ala	10.4	10.7	9.4	8.9	10.0	9.1	8.1	8.0	9.0
Asp	21.4	20.2	15.8	14.6	15.0	16.5	13.5	12.6	12.8
Cys	3.2	4.0	3.4	4.5	4.7	3.3	3.7	4.4	4.2
Glu	38.7	38.1	31.4	29.9	31.7	31.4	26.6	26.6	27.7
Gly	9.0	8.5	8.7	8.8	9.3	7.1	7.9	8.8	8.1
Pro	11.8	11.8	10.5	10.5	11.5	10.1	9.1	9.4	10.4
Ser	9.5	8.8	9.2	8.8	9.4	7.5	8.3	8.4	8.5
Tyr	7.2	7.2	6.0	5.4	6.0	6.0	4.9	4.4	5.2
Gly + Ser	18.5	17.3	17.9	17.6	18.7	14.6	16.2	17.2	16.6
Gly_equi_^2^	15.8	14.8	15.3	15.1	16.0	12.5	13.8	14.8	14.2

1 SP, standard protein diet; RP, reduced-protein; SBM, soybean meal; CM, canola meal; cDDGS, corn distillers’ dried grains with solubles; Gly_equi_, glycine equivalents.

2 Gly_equi_ was calculated using the following equation: concentration of Gly + 0.714 × concentration of Ser.

### Experimental design, birds, and housing

A total of 1,296 male broiler chicks were allocated in a 2 × 4 factorial treatment arrangement (with or without coccidiosis vaccination and four diets). On day 0, birds were assigned to two groups (vaccinated or unvaccinated) in a randomized complete block design with 24 pens (27 birds/pen), with an overall total of 48 pens in the experiment. During the starter phase, data from four adjacent pens were pooled into one data point, yielding 6 replicates (blocks) per treatment (vaccinated vs. unvaccinated). On day 7, each of the vaccination groups was subdivided into four dietary treatments in a split-plot design, resulting in six replicate pens (27 birds/pen) per dietary treatment in both vaccination groups. In the split-plot design, coccidiosis vaccination served as the main plot factor, and dietary treatment served as the subplot factor. The dietary treatments are described in the previous section.

All birds were housed in the same environmentally controlled room, with vaccinated and non-vaccinated groups physically separated on opposite sides of the house by sufficient space. Chickens were raised in clean wood-shaving floor pens, each equipped with nipple drinkers and a single feeder. Temperature, lighting, and ventilation were automatically controlled in accordance with the Cobb Broiler Management Guide (2021). Body weight (**BW**) and feed intake (**FI**) were recorded per pen on days 0, 7, 28, and 42. Mortality rate of birds was used to adjust the calculation of body weight gain (**BWG**), FI, and feed conversion ratio (**FCR**).

### Sample collection

On d 7, two birds per pen were randomly selected and euthanized. Ileal digesta samples collected from the two birds in each of four adjacent pens were pooled to a single sample (8 birds/sample), yielding six replicates per treatment. On d 23, five birds per pen were randomly selected and euthanized to collect the ileal digesta for analysis of AA and titanium, and calculation of AID coefficients. The distal half of the ileum was excised, flushed with distilled water, and pooled within each pen. Jejunal tissues and breast muscle from one of those five birds were collected for quantitative real-time reverse transcriptase polymerase chain reaction (**qRT-PCR**) analysis. On d 23 and 42, two birds per pen were selected for cecal content collection, separately for SCFA and microbiota analyses. Upon sample collection, samples for qRT-PCR and microbiota analyses were rinsed with phosphate-buffered saline, immediately immersed in liquid N, and stored at −80 °C until further analysis. Ileal digesta and cecal contents for SCFA analysis were kept at −20 °C until further analysis.

### Chemical analyses and calculations

The experimental diets and ileal digesta samples were ground to pass through 0.5 mm screen prior to chemical analysis. Dry matter (**DM**) was measured by oven-drying samples according to Method 934.01 (AOAC, 2016). Titanium concentration in the samples was determined as described by Short et al. (1996). The N content was analyzed using the combustion method with a LECO analyzer (LECO Corp., St. Joseph, MI, USA). For AA analysis, samples were hydrolyzed in 6 N hydrochloric acid for 24 h at 100 °C under a N atmosphere according to Method 982.30 E(a) (AOAC, 2016). Prior to hydrolysis, performic acid oxidation was used to determine total sulfur amino acids (**TSAA**), following Method 982.30 E(b) (AOAC, 2016). Quantification was performed using high-performance liquid chromatography after post-column derivatization, according to Method 982.30 E(a, b, and c) (AOAC, 2016).

The coefficient of AID of AA in experimental diets were calculated using the following equation (Yoon and Kong, 2025):

$$\text{AID}\;=\;1\;-\;\frac{{\text{Ti}}_{\text{diet}}}{{\text{Ti}}_{\mathrm{ileal}\;\mathrm{digesta}}}\;\times \;\frac{{\text{AA}}_{\mathrm{ileal}\;\mathrm{digesta}}}{{\text{AA}}_{\text{diet}}},$$

where Ti_diet_ and Ti_ileal digesta_ (g/kg) represent concentrations of titanium in the diet and ileal digesta, respectively; AA_diet_ and AA_ileal digesta_ (g/kg) represent concentrations of AA in the diet and ileal digesta, respectively.

### qRT-PCR analysis

Approximately 100 mg of jejunal and *pectoralis major* (breast muscle) tissues from one bird were homogenized in 1mL of QIAzol Lysis Reagent (Qiagen, Valencia, CA, USA) using 0.5 mm diameter zirconia/silica beads (Biospec Products, Bartlesville, OK, USA). Tissue homogenization was performed for 60 s at 50 × g with a bead beater (Biospec Products Inc., Bartlesville, OK, USA). Total RNA was extracted according to the manufacturer’s protocol, and the RNA pellet was resuspended in 250 μL of HyPure^TM^ Molecular Biology Grade Water (Cytiva, Marlborough, MA, USA). RNA concentration and purity were assessed using the Nanodrop^TM^ Eight Spectrophotometer (Thermo Scientific, Waltham, MA, USA) with 1 μL of the diluted RNA solution. Complementary DNA was synthesized using the High-Capacity Reverse Transcription kit (Applied Biosystems, Foster City, CA, USA). The qRT-PCR was conducted on a QuantStudio 3 Real-Time PCR System (Applied Bioscience, Foster city, CA, USA) using iTaq^TM^ Universal SYBR® Green Supermix (Bio-Rad Laboratories Inc., Waltham, MA, USA). Glyceraldehyde-3-phosphate dehydrogenase was used as the reference gene for normalization. Relative mRNA expression levels in the jejunum and breast muscle tissue were calculated using the 2^−ΔΔCt^ method (Livak and Schmittgen, 2001), with the SP diet in the non-vaccinated group serving as the reference treatment. Primer sequences for all target genes are listed in Table 3.

**Table 3:** Lists of primer sequences used for quantitative real-time reverse transcriptase polymerase chain reaction^1^.

Primers	Sequences	Accession number / Ensembl transcript ID
*GAPDH*	F: CTTTGGCATTGTGGAGGGTC	NM_204305.2
	R: CATCAAAGGTGGAGGAATGG	ENSGALT00010053590.1
*PEPT1*	F: CCCCTGAGGAGGATCACTGTT	NM_204365.2
	R: CAAAAGAGCAGCAGCAACGA	ENSGALT00000027279.6
*B^0,+^AT (SLC7A9)*	F: TTATCACCGCACCTGAAC	NM_001199133.2
	R: AGCATCTGAAGGTGCATAG	ENSGALG00010024831
*CAT1 (SLC7A1)*	F: CCAAGCACGCTGATAAAG	XM_046908303.1
	R: TACTCACAATAGGAAGAAGGG	ENSGALT00010016889.1
*EAAT3 (SLC1A1)*	F: GTGATTGTTCTGAGCGCTGT	XM_046936555.1
	R: ATCCCAGTACCAAAGGCATC	ENSGALT00010028562.1
*OCLDN*	F: ACGGCAGCACCTACCTCAA	XM_046904540.1
	R: GGCGAAGAAGCAGATGAG	ENSGALT00010069214.1
*CLDN1*	F: TGGAGGATGACCAGGTGAAGA	NM_001013611.2
	R: CGAGCCACTCTGTTGCCATA	ENSGALT00010032805.1
*JAM2*	F: AGCCTCAAATGGGATTGGATT	NM_001397141.1
	R: CATCAACTTGCATTCGCTTCA	ENSGALT00010025966.1
*mTOR*	F: TTGGGTTTGCTTTCTGTGGCTGTC	XM_040689168.2
	R: CAGACTTCTGCCTCTTGTGAGCA	ENSGALT00010052862.1
*RPS6KB1*	F: GCCTTTGAAGGTGGAAAACA	XM_046930144.1
	R: TGCTCTGTGCAAGGACTGAC	ENSGALT00010070816.1
*EIF4EBP1*	F: TGAGTGCCTTCCTGTTTCCT	XM_040689367.2
	R: TATTCACACCCACACGGAGA	ENSGALT00010047552.1
*Eef-2*	F: CTGATCAACACCAACCAGACCGA	NM_205368.2
	R: AGCCAATCCAAAGGACCATCCTCA	ENSGALT00010067230.1
*FBXO32*	F: GTGTTGTTCTGCCCATGTTG	NM_001389309.2
	R: CACTGGAGGAAGACGGGATA	ENSGALT00010019724.1
*TRIM36*	F: ATGACCTGAGCTGACCTGAA	NM_018700.4
	R: TTGTCAGGTCAGCTCAGGTC	ENST00000282369.7

1 *GAPDH*, glyceraldehyde-3-phosphorate dehydrogenase; *PEPT1*, peptides transporter 1; *B^0,+^AT*, b (0,+)-type amino acid transporter 1; *CAT1*, cationic amino acid transporter 1; *EAAT3*, excitatory amino acid transporter 3; *OCLDN*, occludin; *CLDN1*, claudin 1; *JAM2*, junctional adhesion molecule 2; *mTOR*, mammalian target of rapamycin; *BPS6KB1*, ribosomal protein S6 kinase beta-1; *EIF4EBP1*, eukaryotic translation initiation factor 4E-binding protein 1; *Eef-2*, eukaryotic elongation factor 2; *FBXO32*, F-box protein 32; *TRIM36*, tripartite motif containing 36.

### Short-chain fatty acids analysis

Samples were analyzed following the procedure described by Lourenco et al. (2020). Approximately 1 g of cecal digesta was diluted with 3 mL of distilled water (1:3 ratio) and centrifuged at 10,000 × g for 10 min. Subsequently, 1 mL of supernatant was mixed with 0.2 mL of 25% meta-phosphoric acid, then frozen overnight. After thawing, the samples were again centrifuged at 10,000 × g for 10 min, and the supernatant was supplemented with an internal standard at a 5:1 ratio (sample: internal standard). The mixture was further diluted with ethyl acetate at a 1:2 ratio, vortexed, and allowed to settle for 5 min. Then, 900 μL of the upper layer was collected and analyzed using gas chromatography (Shimadzu GC-2010 plus; Shimadzu Co., Tokyo, Japan), equipped with a flame ionization detector and a capillary column (Zebron ZB-FFAP; 30 m × 0.32 mm × 0.25 μm; Phenomenex Inc., Torrance, CA, USA). The injection volume was 1.0 μL, with He used as the carrier gas. The oven temperature was initially set at 110°C, gradually increased to 200°C, while the injector and detector temperatures were maintained at 250°C and 350°C, respectively. The SCFA concentrations were determined by comparing the peak heights of the samples to those of standards.

### 16S rRNA amplicon sequencing and analysis

Total genomic DNA was extracted from 0.25 g of cecal digesta preserved in Lysogeny Broth (Becton Dickinson, MD, USA) containing 30% glycerol using the Qiagen DNeasy PowerLyzer PowerSoil kit (Qiagen Inc., MD, USA), following the manufacturer’s protocol (Zwirzitz et al., 2023). Library preparation, quality control, and sequencing of the V3–V4 hypervariable regions of the 16S rRNA gene were performed on the Illumina MiSeq platform.

Paired-end FASTQ files were imported into Quantitative Insights Into Microbial Ecology 2 (**QIIME2**) version 2024.10 for the data processing (Bolyen et al., 2019). Denoising, sequence trimming, and chimera filtering were performed using the DADA2 pipeline. Amplicon sequence variants were taxonomically classified using a Naïve Bayes classifier pretrained on the Greengenes2 database (version 2024.09). Differential abundance analysis was conducted using Linear discriminant analysis Effect Size (**LEfSe**) analysis through the MicrobiomeMaker and Phyloseq packages in RStudio (R version 4.4.1; RStudio PBC, Boston, MA). The significant thresholds were set to 0.05 for the Wilcoxon and Kruskall-Wallis tests, and 3.0 for the linear discriminant analysis (**LDA**) score. To assess the relationship between microbial taxa and SCFAs, a Spearman correlation heatmap was generated using the gplots package in RStudio.

### Statistical analyses

The normality of data was tested using the Shapiro-Wilk test in UNIVARIATE procedure in SAS (SAS Inst., Cary, NC, USA). All data were analyzed using the MIXED procedure in SAS. For data collected on d 7, one-way analysis of variance (**ANOVA**) was performed with vaccination as the fixed effect and replicate as the random effect. For data from the grower and finisher phases, vaccination group was the main-plot factor and dietary treatment was the subplot factor in a split-plot design. Data were analyzed using a two-way ANOVA with the main effects (dietary treatments and vaccination) and their interaction treated as fixed effects, whereas vaccination within block was considered a random effect. Spearman correlation analysis between microbial genera and SCFA was conducted using the CORR procedure in SAS. Least squares means were computed using the LSMEAN statement in SAS. Mean separation was performed using Tukey’s honestly significant difference post-hoc test. The experimental unit was the pen. Statistical significance was set at 0.05, and trends were noted for *P*-values between 0.1 and 0.05.

## Results

### Growth performance

There was no interaction effect between coccidiosis vaccination and dietary treatment on growth performance during the overall experimental period. During the starter phase (d 0-7), coccidiosis vaccination significantly decreased (*P* = 0.042 and 0.041, respectively) the BW on day 7 and BWG of the birds (Table 4). During the grower phase (d 7-28), birds fed SP diet tended (*P* = 0.090) to have greater BWG than other dietary treatments (Table 5). During the finisher phase (d 28-42), no differences in growth performance were observed, except for the BW on d 42, which tended (*P* = 0.064) to be higher in the SP diet group compared to the RP diets. Over the entire experimental period (d 0-42), BWG tended (*P* = 0.064) to be highest in the birds fed the SP diet. There were no treatment effects on FI or FCR throughout the experiment.

**Table 4:** The effect of reduced-crude protein diets and coccidiosis vaccination on growth performance of broilers during the starter stage (d 0 to 7)^1^^,^^2^.

Items	Unvaccinated	Vaccinated	Pooled SE	*P*-value
Day 0 body weight, g	40.9	40.9	0.02	0.294
Body weight gain, g	153	145	2.8	0.041
Feed intake, g	137	136	2.2	0.684
Feed conversion ratio	0.901	0.937	0.0019	0.118

1 SE, standard error.

2 Least squares means represent 6 replicates.

**Table 5:** The effect of reduced-crude protein diets and coccidiosis vaccination on growth performance of broilers from d 7 to 42^1^^,^^2^^,^^3^.

		Body weight, g			Body weight gain, g			Feed intake, g			Feed conversion ratio		
Items		Day 7	Day 28	Day 42	Grower	Finisher	Overall	Grower	Finisher	Overall	Grower	Finisher	Overall
Vaccination	Diet												
Unvaccinated	SP	195	1791	3363	1596	1573	3322	2122	2807	5066	1.330	1.787	1.523
	RP-SBM	198	1759	3268	1561	1508	3227	2090	2840	5064	1.340	1.883	1.570
	RP-CM	197	1739	3331	1541	1592	3290	2148	2850	5137	1.393	1.792	1.562
	RP-cDDGS	184	1762	3355	1578	1592	3314	2150	2720	5006	1.363	1.712	1.510
Vaccinated	SP	190	1779	3392	1588	1613	3351	2125	2889	5146	1.337	1.792	1.535
	RP-SBM	188	1711	3238	1523	1527	3197	2096	2798	5027	1.377	1.840	1.573
	RP-CM	186	1733	3178	1547	1445	3137	2098	2659	4900	1.357	1.842	1.562
	RP-cDDGS	181	1760	3311	1580	1550	3270	2144	2857	5135	1.358	1.848	1.573
Pooled SE (*n* = 6)		5.1	25.7	54.9	23.2	45.4	54.9	24.8	97.1	104.5	0.0185	0.0543	0.0263
Vaccination													
Unvaccinated		194	1763	3329	1569	1566	3288	2127	2804	5068	1.357	1.793	1.541
Vaccinated		186	1746	3280	1560	1534	3239	2116	2801	5052	1.357	1.830	1.561
Pooled SE (*n* = 24)		2.7	12.8	29.7	11.6	25.2	29.7	14.9	48.5	52.4	0.0094	0.0284	0.0136
	**Diet**												
	SP	193	1785	3377	1592	1593	3337	2123	2848	5106	1.333	1.789	1.529
	RP-SBM	193	1735	3253	1542	1518	3212	2093	2819	5046	1.358	1.862	1.572
	RP-CM	192	1736	3254	1544	1518	3213	2123	2755	5018	1.375	1.817	1.562
	RP-cDDGS	183	1761	3333	1579	1571	3292	2147	2788	5071	1.361	1.780	1.542
Pooled SE (*n* = 12)		3.7	18.1	39.9	16.4	33.3	39.9	18.8	68.6	74.0	0.0132	0.0390	0.0188
*P*-values													
Diet		0.145	0.183	0.064	0.090	0.227	0.064	0.153	0.792	0.856	0.140	0.431	0.365
Vaccination		0.042	0.358	0.199	0.567	0.299	0.199	0.489	0.957	0.830	0.904	0.333	0.293
Diet × Vaccination		0.829	0.798	0.401	0.773	0.155	0.401	0.591	0.348	0.319	0.175	0.393	0.589

1 SE, standard error; SBM, soybean meal; CM, canola meal; cDDGS, corn distillers’ dried grains with solubles.

2 SP, standard protein diet containing 200 and 180 g/kg crude protein in the grower and finisher phases, respectively; RP-SBM, reduced-protein corn-SBM-based diet containing 160 and 150 g/kg crude protein in the grower and finisher phases, respectively; RP-CM, RP-SBM diet in which SBM was partially replaced with 80 and 85 g/kg CM in grower and finisher phases, respectively; RP-cDDGS, RP-SBM diet in which SBM was partially replaced with 100 and 110 g/kg cDDGS in grower and finisher phases, respectively.

3 Experimental phases were divided into three separate stages: starter (d 0 to 7), grower (d 7-28), and finisher (d 28-42). The overall period represents the data during the entire experimental period (d 0-42).

### Apparent ileal digestibility of nitrogen and amino acids

On d 7, the coefficient of AID of DM, N, and AA did not differ significantly between vaccination groups (Table 6). However, AID values for all nutrients in vaccinated birds were numerically lower than those in the unvaccinated birds. There was no interaction between vaccination and dietary treatment on AID values on d 23 (Table 7). On d 23, the AID of DM tended (*P* = 0.099) to be higher for birds in the vaccinated group. The AID of N and AA were not significantly different between the vaccinated and unvaccinated groups. However, the AID of N and indispensable AA (His, Ile, Lys, Phe, and Val) in birds fed RP-CM and cDDGS diets was significantly lower (*P* < 0.05) than in the SP diet. In Table 8, birds fed RP-CM and cDDGS diets had a reduced AID of dispensable AA (Asp, Glu, Pro, and Tyr) compared to the SP diet.

**Table 6:** Coefficients of apparent ileal digestibility of dry matter, nitrogen, and amino acids in broilers, vaccinated or unvaccinated against coccidiosis, on d 7.

Items	Unvaccinated	Vaccinated	Pooled SE^1^^,^^2^	*P*-value
Dry matter	0.730	0.714	0.0196	0.578
Nitrogen	0.833	0.807	0.0180	0.338
Indispensable amino acids				
Arg	0.909	0.883	0.0123	0.163
His	0.856	0.829	0.0165	0.278
Ile	0.832	0.804	0.0190	0.332
Leu	0.842	0.814	0.0179	0.285
Lys	0.870	0.834	0.0165	0.155
Met	0.908	0.890	0.0122	0.315
Phe	0.848	0.821	0.0176	0.301
Thr	0.776	0.738	0.0236	0.279
Val	0.813	0.783	0.0208	0.333
Dispensable amino acids				
Ala	0.831	0.800	0.0194	0.293
Asp	0.839	0.812	0.0180	0.323
Cys	0.675	0.627	0.0362	0.375
Glu	0.890	0.867	0.0135	0.246
Gly	0.793	0.756	0.0229	0.280
Pro	0.822	0.795	0.0192	0.350
Ser	0.832	0.798	0.0192	0.240
Tyr	0.847	0.816	0.0176	0.234

1 SE, standard error.

2 Least squares means represent 6 replicates.

**Table 7:** The effect of reduced-crude protein diets and coccidiosis vaccination on coefficients of apparent ileal digestibility of dry matter, nitrogen, and indispensable amino acids in broilers on d 23^1^^,^^2^.

Items		Dry matter	Nitrogen	Arg	His	Ile	Leu	Lys	Met	Phe	Thr	Val
Vaccination	Diet											
Unvaccinated	SP	0.706	0.762	0.871	0.793	0.780	0.771	0.838	0.854	0.789	0.691	0.761
	RP-SBM	0.688	0.725	0.860	0.739	0.742	0.733	0.816	0.848	0.742	0.661	0.728
	RP-CM	0.688	0.718	0.849	0.730	0.722	0.741	0.801	0.866	0.750	0.667	0.729
	RP-cDDGS	0.699	0.719	0.866	0.728	0.755	0.767	0.815	0.862	0.757	0.682	0.730
Vaccinated	SP	0.737	0.787	0.887	0.820	0.809	0.810	0.848	0.891	0.818	0.730	0.792
	RP-SBM	0.749	0.784	0.890	0.799	0.803	0.797	0.848	0.893	0.801	0.741	0.793
	RP-CM	0.718	0.736	0.858	0.745	0.744	0.768	0.800	0.877	0.771	0.690	0.749
	RP-cDDGS	0.708	0.730	0.868	0.739	0.766	0.777	0.813	0.872	0.766	0.700	0.744
Pooled SE (n = 6)		0.0174	0.0180	0.0108	0.0168	0.0180	0.0184	0.0134	0.0129	0.0172	0.0209	0.0182
Vaccination												
Unvaccinated		0.695	0.731	0.861	0.747	0.750	0.753	0.817	0.857	0.759	0.675	0.737
Vaccinated		0.728	0.759	0.876	0.776	0.781	0.788	0.828	0.883	0.789	0.716	0.770
Pooled SE (n = 24)		0.0127	0.0141	0.0092	0.0131	0.0146	0.0143	0.0106	0.0102	0.0136	0.0162	0.0144
	Diet											
	SP	0.722	0.775^a^	0.879^a^	0.807^a^	0.795^a^	0.790	0.843^a^	0.872	0.804^a^	0.711	0.777^a^
	RP-SBM	0.719	0.755^a^^b^	0.875^a^	0.769^b^	0.773^a^^b^	0.765	0.832^a^^b^	0.870	0.771^a^^b^	0.701	0.760^a^^b^
	RP-CM	0.703	0.727^b^	0.853^b^	0.737^b^^c^	0.733^c^	0.755	0.801^c^	0.872	0.761^b^	0.679	0.739^b^
	RP-cDDGS	0.704	0.725^b^	0.867^a^^b^	0.733^c^	0.761^b^^c^	0.772	0.814^b^^c^	0.867	0.762^b^	0.691	0.737^b^
Pooled SE (n = 12)		0.0123	0.0127	0.0076	0.0119	0.0127	0.0130	0.0095	0.0091	0.0122	0.0148	0.0129
*P*-values												
Diet		0.381	0.001	0.002	<0.001	<0.001	0.075	0.001	0.933	0.004	0.204	0.013
Vaccination		0.099	0.192	0.300	0.161	0.162	0.112	0.514	0.104	0.154	0.110	0.138
Diet × Vaccination		0.325	0.280	0.189	0.181	0.212	0.251	0.253	0.141	0.211	0.193	0.219

a-c Means in a column within a group with different superscripts are significantly different (P < 0.05).

1 SE, standard error; SBM, soybean meal; CM, canola meal; cDDGS, corn distillers’ dried grains with solubles.

2 SP, standard protein diet containing 200 and 180 g/kg crude protein in the grower and finisher phases, respectively; RP-SBM, reduced-protein corn-SBM-based diet containing 160 and 150 g/kg crude protein in the grower and finisher phases, respectively; RP-CM, RP-SBM diet in which SBM was partially replaced with 80 and 85 g/kg CM in grower and finisher phases, respectively; RP-cDDGS, RP-SBM diet in which SBM was partially replaced with 100 and 110 g/kg cDDGS in grower and finisher phases, respectively.

**Table 8:** The effect of reduced-crude protein diets and coccidiosis vaccination on the coefficients of apparent ileal digestibility of dispensable amino acids in broilers on d 23^1^^,^^2^.

Items		Ala	Asp	Cys	Glu	Gly	Pro	Ser	Tyr
Vaccination	Diet								
Unvaccinated	SP	0.762	0.779	0.685	0.831	0.732	0.753	0.763	0.788
	RP-SBM	0.725	0.724	0.693	0.799	0.737	0.703	0.769	0.732
	RP-CM	0.736	0.708	0.722	0.804	0.749	0.696	0.769	0.699
	RP-cDDGS	0.751	0.701	0.717	0.805	0.726	0.727	0.773	0.750
Vaccinated	SP	0.802	0.798	0.723	0.856	0.759	0.789	0.793	0.829
	RP-SBM	0.792	0.782	0.769	0.844	0.796	0.773	0.823	0.793
	RP-CM	0.761	0.722	0.743	0.820	0.762	0.720	0.783	0.736
	RP-cDDGS	0.764	0.705	0.724	0.809	0.732	0.737	0.781	0.765
Pooled SE (n = 6)		0.0190	0.0184	0.0187	0.0143	0.0171	0.0188	0.0150	0.0191
Vaccination									
Unvaccinated		0.743	0.728	0.704	0.810	0.736	0.720	0.768	0.742
Vaccinated		0.780	0.752	0.740	0.832	0.762	0.755	0.795	0.781
Pooled SE (n = 24)		0.0147	0.0146	0.0141	0.0114	0.0133	0.0143	0.0118	0.0157
	Diet								
	SP	0.782	0.789^a^	0.704	0.843^a^	0.745^a^^b^	0.771^a^	0.778	0.809^a^
	RP-SBM	0.759	0.753^b^	0.731	0.821^a^^b^	0.766^a^	0.738^a^^b^	0.796	0.762^b^
	RP-CM	0.748	0.715^c^	0.733	0.812^b^	0.755^a^^b^	0.708^b^	0.776	0.718^c^
	RP-cDDGS	0.757	0.703^c^	0.720	0.807^b^	0.729^b^	0.732^b^	0.777	0.757^b^
Pooled SE (n = 12)		0.0135	0.0130	0.0133	0.0101	0.0121	0.0133	0.0106	0.0135
*P*-values									
Diet		0.118	<0.001	0.192	0.006	0.036	0.001	0.210	<0.001
Vaccination		0.112	0.278	0.106	0.197	0.188	0.113	0.139	0.111
Diet × Vaccination		0.255	0.198	0.119	0.232	0.174	0.199	0.148	0.351

a-c Means in a column within a group with different superscripts are significantly different (P < 0.05).

1 SE, standard error; SBM, soybean meal; CM, canola meal; cDDGS, corn distillers’ dried grains with solubles.

2 SP, standard protein diet containing 200 and 180 g/kg crude protein in the grower and finisher phases, respectively; RP-SBM, reduced-protein corn-SBM-based diet containing 160 and 150 g/kg crude protein in the grower and finisher phases, respectively; RP-CM, RP-SBM diet in which SBM was partially replaced with 80 and 85 g/kg CM in grower and finisher phases, respectively; RP-cDDGS, RP-SBM diet in which SBM was partially replaced with 100 and 110 g/kg cDDGS in grower and finisher phases, respectively.

### Relative expressions of peptide and amino acids transporters, tight junction proteins, and protein metabolism-related genes

No interaction effects between vaccination and dietary treatment were observed on peptide and AA transporters in the jejunum tissue (Table 9). The vaccinated birds tended to have greater (*P* = 0.054) relative *peptide transporter 1* relative gene expression on d 23. An interaction effect (*P* = 0.029) was observed for tight junction protein *occludin* (***OCLDN***), where birds fed the SP diet had greater jejunal *OCLDN* expression compared to the RP-CM diet in the vaccinated group, while no differences were found in the unvaccinated group.

**Table 9:** The effect of reduced-crude protein diets and coccidiosis vaccination on relative gene expressions of peptide and amino acid transporters and tight junction proteins in jejunum tissue of broilers on d 23^1^^,^^2^.

		Peptide and amino acids transporters				Tight junction proteins		
Items		*PEPT1*	*B^0,+^AT*	*CAT1*	*EAAT3*	*OCLDN*	*CLDN1*	*JAM2*
Vaccination	Diet							
Unvaccinated	SP	1.00	1.00	1.00	1.00	1.00^a^^b^	1.00	1.00
	RP-SBM	1.10	1.04	0.83	0.66	1.04^a^^b^	0.88	0.83
	RP-CM	1.14	1.17	1.13	0.84	1.08^a^^b^	1.02	1.40
	RP-cDDGS	1.14	1.08	0.87	0.90	1.00^a^^b^	0.86	1.06
Vaccinated	SP	3.15	1.84	1.56	1.50	1.72^a^	1.81	2.75
	RP-SBM	1.85	1.51	1.15	1.06	1.07^a^^b^	1.09	1.54
	RP-CM	1.35	1.42	0.82	1.07	0.64^b^	0.86	0.75
	RP-cDDGS	1.10	0.81	1.00	0.97	1.03^a^^b^	0.88	1.01
Pooled SE (n = 6)		0.548	0.439	0.263	0.378	0.184	0.291	0.625
Vaccination								
Unvaccinated		1.10	1.07	0.96	0.85	1.03	0.94	1.07
Vaccinated		1.87	1.40	1.13	1.15	1.11	1.16	1.51
Pooled SE (n = 24)		0.274	0.221	0.147	0.195	0.092	0.146	0.313
	Diet							
	SP	2.08	1.42	1.28	1.25	1.36	1.41	1.88
	RP-SBM	1.48	1.28	0.99	0.86	1.05	0.99	1.19
	RP-CM	1.24	1.30	0.97	0.95	0.86	0.94	1.07
	RP-cDDGS	1.12	0.95	0.94	0.93	1.02	0.87	1.04
Pooled SE (n = 12)		0.388	0.311	0.193	0.270	0.130	0.206	0.442
*P*-values								
Diet		0.323	0.734	0.503	0.737	0.067	0.263	0.506
Vaccination		0.054	0.301	0.326	0.268	0.517	0.283	0.324
Diet × Vaccination		0.205	0.649	0.374	0.941	0.029	0.383	0.266

a-b Means in a column within a group with different superscripts are significantly different (P < 0.05).

1 *PEPT1*, peptides transporter 1; *B^0,+^AT*, b (0,+)-type amino acid transporter 1; *CAT1*, cationic amino acid transporter 1; *EAAT3*, excitatory amino acid transporter 3; *OCLDN*, occludin; *CLDN1*, claudin 1; *JAM2*, junctional adhesion molecule 2; SE, standard error; SBM, soybean meal; CM, canola meal; cDDGS, corn distillers’ dried grains with solubles.

2 SP, standard protein diet containing 200 and 180 g/kg crude protein in the grower and finisher phases, respectively; RP-SBM, reduced-protein corn-SBM-based diet containing 160 and 150 g/kg crude protein in the grower and finisher phases, respectively; RP-CM, RP-SBM diet in which SBM was partially replaced with 80 and 85 g/kg CM in grower and finisher phases, respectively; RP-cDDGS, RP-SBM diet in which SBM was partially replaced with 100 and 110 g/kg cDDGS in grower and finisher phases, respectively.

No interaction effects were detected for the relative gene expression of protein synthesis and degradation in the breast muscle (Table 10). The relative expression of the protein synthesis gene *ribosomal protein S6 kinase B1* (***RPS6KBI***) differed significantly (*P* = 0.049) between dietary treatment, with birds fed the SP diet showing the highest *RPS6KBI* expression among the diets. Another protein synthesis gene *eukaryotic translation initiation factor 4E-binding protein 1* (***EIF4EBP1***) showed greater expression (*P* = 0.046) in the unvaccinated group compared to the vaccinated group.

**Table 10:** The effect of reduced-crude protein diets and coccidiosis vaccination on relative expression of protein synthesis and degradation genes in pectoralis major breast muscle of broilers on d 23^1^^,^^2^.

		Protein synthesis				Protein degradation	
Items		*mTOR*	*RPS6KB1*	*EIF4EBP1*	*Eef-2*	*FBXO32*	*TRIM36*
Vaccination	Diet						
Unvaccinated	SP	1.00	1.00	1.00	1.00	1.00	1.00
	RP-SBM	0.85	0.80	0.83	0.86	0.83	0.88
	RP-CM	0.80	0.85	0.77	0.79	0.64	0.66
	RP-cDDGS	1.08	1.03	0.96	0.98	0.65	0.69
Vaccinated	SP	1.28	1.19	0.81	1.04	0.64	0.73
	RP-SBM	0.77	0.79	0.63	0.70	0.58	0.63
	RP-CM	0.95	0.98	0.75	0.93	0.75	0.82
	RP-cDDGS	1.09	1.06	0.72	0.90	1.26	1.31
Pooled SE (n = 6)		0.164	0.113	0.110	0.133	0.234	0.235
Vaccination							
Unvaccinated		0.93	0.92	0.89	0.91	0.78	0.81
Vaccinated		1.02	1.00	0.73	0.89	0.81	0.88
Pooled SE (n = 24)		0.082	0.056	0.055	0.066	0.123	0.124
	Diet						
	SP	1.14	1.09^a^	0.90	1.02	0.82	0.87
	RP-SBM	0.81	0.80^a^	0.73	0.78	0.70	0.75
	RP-CM	0.87	0.91^a^	0.76	0.86	0.70	0.74
	RP-cDDGS	1.08	1.05^a^	0.84	0.94	0.95	1.00
Pooled SE (n = 12)		0.116	0.080	0.077	0.094	0.168	0.169
*P*-values							
Diet		0.146	0.049	0.387	0.320	0.646	0.648
Vaccination		0.445	0.296	0.046	0.868	0.861	0.680
Diet × Vaccination		0.711	0.812	0.756	0.700	0.161	0.191

a Means in a column within a group with different superscripts are significantly different (P < 0.05).

1 *mTOR*, mammalian target of rapamycin; *BPS6KB1*, ribosomal protein S6 kinase beta-1; *EIF4EBP1*, Eukaryotic translation initiation factor 4E-binding protein 1; *Eef-2*, eukaryotic elongation factor 2; *FBXO32*, F-box protein 32; *TRIM36*, tripartite motif containing 36; SE, standard error; SBM, soybean meal; CM, canola meal; cDDGS, corn distillers’ dried grains with solubles.

2 SP, standard protein diet containing 200 and 180 g/kg crude protein in the grower and finisher phases, respectively; RP-SBM, reduced-protein corn-SBM-based diet containing 160 and 150 g/kg crude protein in the grower and finisher phases, respectively; RP-CM, RP-SBM diet in which SBM was partially replaced with 80 and 85 g/kg CM in grower and finisher phases, respectively; RP-cDDGS, RP-SBM diet in which SBM was partially replaced with 100 and 110 g/kg cDDGS in grower and finisher phases, respectively.

### Cecal short-chain fatty acids profiles

There were no significant interactions between vaccination and dietary treatment on cecal SCFA profiles on d 23 (Table 11). The birds fed RP-CM diet (*P* < 0.05) reduced cecal propionate and isobutyrate concentrations (*P* < 0.05) compared to the SP diet. The birds fed RP-SBM diet significantly decreased (*P* < 0.05) cecal acetate concentration, consequently significantly lowering (*P* < 0.05) the concentrations of unbranched-chain fatty acids and total SCFA profiles compared to the SP diet.

**Table 11:** The effect of reduced-crude protein diets and coccidiosis vaccination on short-chain fatty acid (SCFA, mM) profiles of broilers on d 23^1^^,^^2^^,^^3^.

Items		Acetate	Propionate	Isobutyrate	Butyrate	Isovalerate	Valerate	UBCFA	BCFA	Total SCFA
Vaccination	Diet									
Unvaccinated	SP	87.2	6.36	0.68	18.7	0.61	1.25	113.5	1.29	114.8
	RP-SBM	66.6	4.25	0.68	17.0	0.65	1.15	89.0	1.83	90.3
	RP-CM	75.3	3.47	0.48	15.1	0.47	1.28	95.2	1.33	96.2
	RP-cDDGS	70.6	4.57	0.57	12.6	0.41	1.21	89.1	1.22	90.0
Vaccinated	SP	83.2	6.75	0.98	16.8	0.85	1.28	108.1	0.96	109.9
	RP-SBM	69.1	5.08	0.66	11.8	0.55	1.09	87.1	0.82	88.4
	RP-CM	72.4	3.27	0.47	14.6	0.36	0.95	91.2	0.98	92.0
	RP-cDDGS	86.5	5.43	0.92	15.3	0.85	1.33	108.6	1.77	110.4
Pooled SE (n = 6)		6.25	0.770	0.134	2.22	0.137	0.140	8.32	0.268	8.35
Vaccination										
Unvaccinated		74.9	4.66	0.60	15.9	0.53	1.22	96.7	1.14	97.8
Vaccinated		77.8	5.14	0.76	14.6	0.65	1.16	98.8	1.41	100.2
Pooled SE (n=24)		3.40	0.415	0.075	1.16	0.074	0.082	4.63	0.149	4.71
	Diet									
	SP	85.2^a^	6.56^a^	0.83^a^	17.7	0.73	1.26	110.8^a^	1.56	112.3^a^
	RP-SBM	67.8^b^	4.67^a^^b^	0.67^a^^b^	14.4	0.60	1.12	88.1^b^	1.27	89.3^b^
	RP-CM	73.9^a^^b^	3.37^b^	0.47^b^	14.8	0.42	1.11	93.2^a^^b^	0.89	94.1^a^^b^
	RP-cDDGS	78.6^a^^b^	5.00^a^^b^	0.74^a^^b^	14.0	0.63	1.27	98.8^a^^b^	1.37	100.2^a^^b^
Pooled SE (n=12)		4.42	0.544	0.095	1.57	0.097	0.099	5.89	0.189	5.90
*P*-values										
Diet		0.049	0.002	0.058	0.321	0.146	0.464	0.047	0.088	0.041
Vaccination		0.563	0.439	0.182	0.476	0.285	0.605	0.758	0.227	0.732
Diet × Vaccination		0.350	0.882	0.329	0.362	0.127	0.370	0.373	0.202	0.347

a-b Means in a column within a group with different superscripts are significantly different (P < 0.05).

1 UBCFA, unbranched-chain fatty acids; BCFA, branched-chain fatty acids; SE, standard error; SBM, soybean meal; CM, canola meal; cDDGS, corn distillers’ dried grains with solubles.

2 SP, standard protein diet containing 200 and 180 g/kg crude protein in the grower and finisher phases, respectively; RP-SBM, reduced-protein corn-SBM-based diet containing 160 and 150 g/kg crude protein in the grower and finisher phases, respectively; RP-CM, RP-SBM diet in which SBM was partially replaced with 80 and 85 g/kg CM in grower and finisher phases, respectively; RP-cDDGS, RP-SBM diet in which SBM was partially replaced with 100 and 110 g/kg cDDGS in grower and finisher phases, respectively.

3 UBCFA is the sum of acetate, propionate, butyrate, and valerate; BCFA is the sum of isobutyrate and isovalerate; total SCFA is the sum of acetate, propionate, isobutyrate, butyrate, isovalerate, and valerate.

No interaction effects were found on cecal SCFA concentrations on d 42 (Table 12). Birds fed the RP-cDDGS diet exhibited a significantly greater (*P* < 0.05) isobutyrate concentration compared to those fed the SP diet. The cecal isovalerate concentration tended to be greater (*P* = 0.058) for birds fed the RP-cDDGS diet compared to the other treatments. Consequently, the concentration of branched-chain fatty acids (**BCFA**) in cecal content was significantly greater (*P* < 0.05) in birds fed the RP-cDDGS diet compared to the SP diet.

**Table 12:** The effect of reduced-crude protein diets and coccidiosis vaccination on short-chain fatty acid (SCFA, mM) profiles of broilers on d 42^1^^,^^2^^,^^3^.

Items		Acetate	Propionate	Isobutyrate	Butyrate	Isovalerate	Valerate	UBCFA	BCFA	Total SCFA
Vaccination	Diet									
Unvaccinated	SP	86.6	10.76	0.89	16.4	0.82	1.40	115.2	1.71	116.9
	RP-SBM	75.9	7.85	0.93	13.5	0.86	1.35	98.6	1.79	100.4
	RP-CM	73.1	4.40	0.85	11.1	0.67	1.14	89.7	1.52	91.2
	RP-cDDGS	85.8	8.37	1.42	12.7	1.15	1.66	108.5	2.57	111.1
Vaccinated	SP	69.6	7.24	0.56	12.6	0.48	1.06	90.5	1.04	91.6
	RP-SBM	70.9	7.54	1.02	11.4	0.83	1.18	91.0	1.85	92.8
	RP-CM	79.5	7.85	0.91	13.4	0.75	1.31	102.0	1.66	103.7
	RP-cDDGS	68.7	5.68	1.06	12.6	0.98	1.36	88.4	2.04	90.4
Pooled SE (n = 6)		8.08	1.995	0.143	2.09	0.159	0.165	10.34	0.297	10.33
Vaccination										
Unvaccinated		80.3	7.85	1.02	13.4	0.88	1.39	103.0	1.90	104.9
Vaccinated		72.2	7.08	0.89	12.5	0.76	1.23	93.0	1.65	94.6
Pooled SE (n = 24)		4.04	1.014	0.071	1.05	0.080	0.083	5.17	0.149	5.17
	Diet									
	SP	78.1	9.00	0.73^b^	14.5	0.65	1.23	102.9	1.38^b^	104.2
	RP-SBM	73.4	7.69	0.98^a^^b^	12.4	0.84	1.26	94.8	1.82^a^^b^	96.6
	RP-CM	76.3	6.13	0.88^a^^b^	12.3	0.71	1.22	95.9	1.59^a^^b^	97.5
	RP-cDDGS	77.3	7.03	1.24^a^	12.6	1.07	1.51	98.4	2.31^a^	100.7
Pooled SE (n = 12)		5.72	1.411	0.101	1.48	0.113	0.117	7.31	0.210	7.31
*P*-values										
Diet		0.942	0.536	0.008	0.690	0.058	0.258	0.868	0.021	0.878
Vaccination		0.162	0.590	0.202	0.521	0.298	0.187	0.178	0.242	0.167
Diet × Vaccination		0.419	0.314	0.239	0.512	0.595	0.397	0.295	0.428	0.276

a-b Means in a column within a group with different superscripts are significantly different (P < 0.05).

1 UBCFA, unbranched-chain fatty acids; BCFA, branched-chain fatty acids; SE, standard error; SBM, soybean meal; CM, canola meal; cDDGS, corn distillers’ dried grains with solubles.

2 SP, standard protein diet containing 200 and 180 g/kg crude protein in the grower and finisher phases, respectively; RP-SBM, reduced-protein corn-SBM-based diet containing 160 and 150 g/kg crude protein in the grower and finisher phases, respectively; RP-CM, RP-SBM diet in which SBM was partially replaced with 80 and 85 g/kg CM in grower and finisher phases, respectively; RP-cDDGS, RP-SBM diet in which SBM was partially replaced with 100 and 110 g/kg cDDGS in grower and finisher phases, respectively.

3 UBCFA is the sum of acetate, propionate, butyrate, and valerate; BCFA is the sum of isobutyrate and isovalerate; total SCFA is the sum of acetate, propionate, isobutyrate, butyrate, isovalerate, and valerate.

### Cecal microbiota

**Fig. 1:**
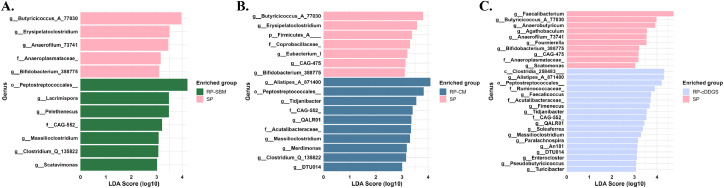
Differentially expressed amplicon sequence variants (ASVs) at the genus level in the cecal microbiota of 42-day-old broiler chickens fed the SP diet, compared with those fed the RP-SBM (A), RP-CM (B), or RP-cDDGS (C) diet, as identified by Linear Discriminant Analysis Effect Size analysis. Different colors represent different dietary treatments (n = 12). The ASVs were classified at the phylum, family, and order levels based on their genus-level identity. Abbreviations: SBM, soybean meal; CM, canola meal; cDDGS, corn distillers’ dried grains with solubles; LDA, linear discriminant analysis; SP, standard protein diet containing 200 and 180 g/kg crude protein in the grower and finisher phases, respectively; RP-SBM, reduced-protein corn-SBM-based diet containing 160 and 150 g/kg crude protein in the grower and finisher phases, respectively; RP-CM, RP-SBM diet in which SBM was partially replaced with 80 and 85 g/kg CM in grower and finisher phases, respectively; RP-cDDGS, RP-SBM diet in which SBM was partially replaced with 100 and 110 g/kg cDDGS in grower and finisher phases, respectively.

Differentially expressed microbes at the genus level between birds fed SP and RP diets on d 42 were analyzed using the LEfSe analysis (Fig. 1). In comparison between birds fed SP and RP-SBM diets, the SP diet group was associated with greater abundance of genera *Butyricicoccus A 77030, Anaerofilum 73741, Erysipelatoclostridium, Bifidobacterium 388775*, and an unclassified family *Anaeroplasmataceae*. Conversely, the SP-SBM group exhibited an enriched abundance of genera *Pelethenecus, Lacrimispora, Scatavimonas, Massilioclostridium, Clostridium Q 135822*, an unclassified family *CAG-552*, and an unclassified order *Peptostreptococcales*.

Between birds fed SP and RP-CM diets, the SP diet group exhibited greater abundance of genera *Butyricicoccus A 77030, Erysipelatoclostridium, Eubacterium I, CAG-475, Bifidobacterium 388775,* an unclassified family *Coprobacillaceae,* and an unclassified phylum *Firmicutes A.* In contrast, birds fed the RP-CM diet showed increased abundance of genera *Alistipes A 871400, Tidjanibacter, QALR01, Massilioclostridium, Merdimonas, Clostridium Q 135822, DTU014,* unclassified families *Acutalibacteraceae* and *CAG-552*, and an unclassified order *Peptostreptococcales*.

Similarly, the SP diet group had a higher abundance of genera *Faecalibacterium, Anaerobutyricum, Butyricicoccus A 77030, Agathobaculum, Fournierella, Anaerofilum 73741, CAG-475, Bifidobacterium 388775,* and an unclassified family *Anaeroplasmataceae* compared to the RP-cDDGS diet. Conversely, birds fed the RP-cDDGS diet had enriched abundance of genera *Alistipes A 871400, Faecalicoccus, Fimenecus, Tidjanibacter, QALR01, Soleaferrea, Massilioclostridium, Turicibacter, Enterocloster, DTU014, Paralachnospira, Pseudobutyricicoccus, An181*, unclassified families *Ruminococcaceae, Acutalibacteraceae, CAG-552*, an unclassified order *Peptostreptococcales*, and unclassified class *Clostridia 258483*.

**Fig. 2:**
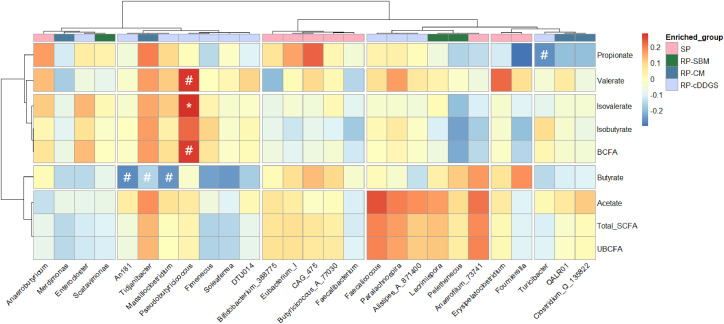
Heatmap of Spearman correlation analysis between short-chain fatty acids (SCFAs) and differentially expressed genera (amplicon sequence variants) in the cecal microbiota of 42-day-old broiler chickens. Birds were fed either a SP diet or RP diets containing SBM, CM, or cDDGS (n = 48). Abbreviations: UBCFA, unbranched-chain fatty acids, BCFA, branched-chain fatty acids; SBM, soybean meal; CM, canola meal; cDDGS, corn distillers’ dried grains with solubles; SP, standard protein diet containing 200 and 180 g/kg crude protein in the grower and finisher phases, respectively; RP-SBM, reduced-protein corn-SBM-based diet containing 160 and 150 g/kg crude protein in the grower and finisher phases, respectively; RP-CM, RP-SBM diet in which SBM was partially replaced with 80 and 85 g/kg CM in grower and finisher phases, respectively; RP-cDDGS, RP-SBM diet in which SBM was partially replaced with 100 and 110 g/kg cDDGS in grower and finisher phases, respectively; *, P < 0.05; #, 0.05 < P < 0.1.

The SCFA profiles in the ceca are considered to be closely correlated with the cecal microbiome; therefore, Spearman correlation analysis was performed to investigate the relationship between differentially expressed microbes and cecal SCFA profile (Fig. 2). Significant (*P* < 0.05) or trending (*P* < 0.10) correlations were observed in birds fed the RP-CM and cDDGS diets. Butyrate concentrations in the cecal digesta tended (*P* < 0.10) to negatively correlate with genera *An181, Tidjanibacter, Massilioclostridium* in birds fed the RP-CM and RP-cDDGS diets. Propionate concentration tended (P < 0.10) to negatively correlate with genus *Turicibacter* in birds fed the RP-cDDGS diet. The genus *Pseudobutyricicoccus* exhibited a significant (*P* < 0.05) positive correlation with isovalerate and tendencies (*P* < 0.10) for positive correlation with valerate and BCFA in the cecal content of birds fed the RP-cDDGS diet.

## Discussion

Most RP diet studies have traditionally been conducted using corn–SBM–based diets (Lee et al., 2022; Lee et al., 2011). There is still a paucity of research on RP diets in broiler chickens in which alternative protein sources, such as CM and cDDGS, are partially replaced with SBM. The present findings may provide a practical reference for the feed industry, particularly in contexts where protein sources other than SBM are available. The primary objective of the present study was to investigate whether SBM could be partially replaced with CM or cDDGS in a RP corn-SBM-based diet for broiler chickens following coccidiosis vaccination at placement. Our previous studies reported that the partial inclusion of CM and cDDGS had a negative impact on the growth performance of broilers under normal rearing conditions, and on their immune responses under *Eimeria*-challenged conditions (Ajao et al., 2022; Shanmugasundaram et al., 2024). Building upon these findings, we hypothesized that the use of CM and cDDGS in RP diets at the expense of SBM would impair growth performance, nutrient digestibility, and intestinal metabolite profiles, as well as alter the microbial composition, particularly in broilers vaccinated against coccidiosis.

### Growth performance

In the present study, reductions in BWG and BW on d 7 were observed in vaccinated birds during the starter phase. Das et al. (2021) reported growth retardation in vaccinated broilers against coccidiosis between d 10 and 20; however, their subsequent growth performance was comparable to that of unvaccinated birds. Similarly, Orso et al. (2022) observed a reduction in BWG in coccidiosis-vaccinated broilers from d 1−21, with no adverse effects in the later phases. These findings, together with the present results, suggest that the cycling of the coccidiosis vaccine predominantly occurs during the early stages of age, with its negative impact on growth performance diminishing by approximately 3 weeks of age in broilers. Nevertheless, previous literature has reported inconsistent results, with some studies seeing no significant impact of coccidiosis vaccination on growth performance of broilers during the starter phase (da Silva Giacomini et al., 2023; Gautier et al., 2019). These discrepancies might be due to variations in the composition of *Eimeria* species included in the vaccines, differences in vaccinal strains, or different vaccine dosages used across studies (Zaheer et al., 2022).

No significant differences in growth performance were observed between vaccinated and unvaccinated broilers during the subsequent phases (d 7−42). However, growth performance tended to differ depending on the dietary treatment. Specifically, birds fed the SP diet tended to exhibit higher BWG throughout the entire experimental period and greater cumulative BW on d 42 compared with those receiving the RP diets. This observation may be attributed to the limitation of certain AA in the RP diets. Dean et al. (2006) demonstrated that dietary glycine can become limiting when birds are fed the RP diets, and that supplemental glycine optimizes the growth performance of broilers. Glycine is considered a semi-essential AA, as it plays a crucial role in the Krebs uric acid cycle involved in uric acid excretion (Patience, 1990). Given the interconvertible nature of Gly and Ser, the total Gly + Ser concentrations are often expressed as Gly_equi_ (Siegert et al., 2015). In RP diets, reduced inclusion rate of SBM leads to decreased dietary concentrations of dispensable AAs, necessitating careful monitoring of Gly_equi_ levels to prevent potential negative effects on growth performance of broilers. In the present study, the dietary digestible Gly_equi_ concentrations in the RP diets were maintained at 12.0 and 11.3 g/kg during the grower and finisher phases, respectively. Correspondingly, the analyzed total Gly + Ser concentrations in the RP diets were above 17.6 g/kg during the grower phase and 16.2 g/kg during the finisher phase. These values exceeded those reported by Lee et al. (2022), who observed no significant differences in BW of broilers when dietary CP concentrations were reduced by 25 g/kg (grower) and 30 g/kg (finisher), provided that dietary total Gly + Ser concentrations were maintained at 16.6 g/kg (grower) and 13.5 g/kg (finisher), respectively. Therefore, the tendency of reductions in BW at day 42 and in BWG during the later phases observed in the present study is unlikely to be solely attributable to insufficient total Gly + Ser concentrations. Supporting this, Ajao et al. (2022) reported that a 50 g/kg reduction in dietary CP concentration impaired growth performance compared to the SP diet, despite similar Gly_equi_ concentrations to those used in the present study. This suggests that the impaired growth performance may stem from deficiencies in other dispensable AAs, rather than Gly or Ser alone. Substantial reductions in dietary CP concentrations (exceeding 4%) inherently lead to decreased concentrations of overall dispensable AA in the diet, which may, in turn, adversely affect the growth performance of broiler chickens.

Another plausible contributing factor is the interaction between TSAA and total Gly + Ser requirements. Specifically, Ser is required for Cys synthesis from Met (Siegert et al., 2015). Siegert et al. (2015) reported that the optimal Met:TSAA ratio for broiler chickens is 0.65, while a lower ratio (0.50) resulted in reduced average daily gain. In the present study, the Met:Cys ratio was 0.52 in the experimental grower and finisher diets, which may have adversely affected the growth performance of broiler chickens, particularly in the RP diets where sufficient concentrations of total Gly + Ser are critical. To conclude, the findings of the present study suggest that the effects of dietary RP are primarily attributable to the reduction in dietary CP concentration, rather than to the differences in protein sources included in the RP diet.

### Apparent ileal digestibility of nitrogen and amino acids

The observed numerical reduction in the AID of DM and AA in the vaccinated group during the starter phase in the present study aligns with previous findings. Gautier and Rochell (2020) reported that coccidiosis vaccination reduced the AID of N and ether extract in broiler chickens fed corn-SBM-based diets at day 12 post-hatch compared to unvaccinated controls. Similarly, Myers and Rochell (2024) demonstrated that broilers subjected to a coccidiosis vaccination program exhibited reduced AID of DM, N, ether extract, and energy, as well as reduced ileal digestible energy concentrations, compared to unvaccinated birds that received an anticoccidial drug at day 11 post-hatch. These reductions in nutrient digestibility are likely due to active coccidiosis cycling during the early phase of growth. As such, the compromised digestion and absorption of AA may have contributed to the impaired growth performance observed in vaccinated birds during the early stages of the present study.

On d 23, the AID of most AAs was lower in the RP diets containing CM and cDDGS compared to the SP diet. This reduction may be explained by the higher IDF content present in CM and cDDGS relative to the SBM (Acosta et al., 2020; Navarro et al., 2018), but this effect was independent of whether the birds were vaccinated or not. High levels of IDF can physically entrap nutrients, limiting their exposure to digestive enzymes in the gastrointestinal tract and thereby decreasing nutrient digestibility. Consequently, the tendency of reduced BWG observed in birds fed RP diets including CM and cDDGS during the grower phase may, in part, also result from the reduced AID of AAs in these experimental diets.

In contrast to the finding of the present study, our previous study reported that 21-day-old broilers fed corn-SBM-based RP diet with a 140 g/kg CP exhibited higher AID of AA compared to the SP diet containing 180 g/kg CP (Ajao and Olukosi, 2024). This discrepancy may be due to the more pronounced CP reduction (up to 140 g/kg CP) in the RP diet of previous study compared with the present study, which necessitated a greater dietary inclusion of crystalline AA and may have consequently increased the AID of AA.

### Relative expressions of peptide and amino acid transporters, tight junction proteins, and protein metabolism-related genes

The increased relative expression of the jejunal peptide transporter *PEPT1* in vaccinated birds at day 23 supports the observed improvement in AID of AA in the present study. In contrast to our finding, Ajao and Olukosi (2024) reported a decreased expression of PEPT1 in broilers fed a corn-SBM-based RP diet containing 140 g/kg CP, compared to those fed an SP diet containing 180 g/kg CP. This may be attributed to the reduced intake of intact protein in the RP diet, which could diminish the need for gastric digestion, in turn, lower the demand for peptide transporter expression.

Within the vaccinated group, birds fed the RP-CM diet exhibited the lowest relative expression of *OCLDN* among all dietary treatments. This finding suggests that CM may impair tight junction protein integrity, likely due to its physical characteristics, particularly in birds vaccinated against coccidiosis, and may ultimately compromise gut health of birds. Additionally, the reduced expression of the protein synthesis gene *EIF4EBP1* implies that vaccinated birds may prioritize the repair and maintenance of the gastrointestinal tract over protein synthesis. This trade-off aligns with the well-documented impact of immune system activation on growth performance, which is primarily mediated through the nutrient redirection toward immune responses at the expense of growth and feed efficiency (Rauw, 2012). Similarly, Shanmugasundaram et al. (2024) demonstrated that *Eimeria*-challenged broilers exhibited greater CD4+ T cell populations and nitric oxide production compared to uninfected birds, suggesting an increased immune burden.

### Cecal short-chain fatty acid profiles and microbiota

Greater intake of IDF could shorten the retention time of intestinal digesta and reduce microbial fermentation, subsequently decreasing fiber availability and SCFA production in the hindgut (Zhang et al., 2023). The alternative protein sources, CM and cDDGS, generally contain approximately twice the level of IDF compared to SBM (Acosta et al., 2020; Navarro et al., 2018). Therefore, it was hypothesized that RP-CM and cDDGS diets would lead to reduced fermentation and lower SCFA concentrations in the hindgut. In line with this hypothesis, birds fed the RP-CM diet exhibited decreased cecal propionate and isobutyrate concentrations compared to those fed the SP diet on day 23. Similarly, Konieczka et al. (2019) reported that partial replacement of SBM with rapeseed meal in a corn-SBM-based broiler diet reduced total SCFA concentrations in the ileal and cecal digesta. Unexpectedly, the RP-SBM diet also resulted in reduced acetate concentration, which contributed to the lowest total SCFA concentrations among the dietary treatments, as acetate accounts for more than 70% of the total SCFA profile in the present study. Although the underlying mechanism of this observation remains unclear, it may be associated with limited fermentable fiber availability. This is likely attributable to the relatively low fiber content of SBM, and reductions in dietary SBM inclusion further decrease the fiber content of the diet.

The cecal SCFA profile on d 42 differed markedly from that observed on d 23. The differences might be attributed to the birds’ adaptation to the experimental diets and an increased ability to ferment fiber and protein with age. Notably, broilers fed the RP-cDDGS diets had the highest BCFA concentrations, including isobutyrate and isovalerate. It is well documented that increased BCFA production is indicative of greater protein fermentation, leading to the production of potentially harmful metabolites such as ammonia, sulfur-containing compounds, biogenic amines, and phenols, all of which may negatively impact intestinal health (Qaisrani et al., 2015). The elevated cecal BCFA concentrations observed in birds fed the RP-cDDGS diet may be due to higher concentrations of ileal indigestible protein (indirectly estimated using N values), with values of 15.5, 14.6, 16.8, and 17.6 g/kg for SP, RP-SBM, RP-CM, and RP-cDDGS diets, respectively, that likely reached the ceca. Therefore, although cecal protein concentrations were not measured in the present study, the increased passage of undigested protein into the hindgut in birds fed the RP-cDDGS diet might have increased the ratio of protein to fermentable fiber, thereby shifting microbial fermentation toward protein rather than fiber; however, this remains a supposition.

Coccidiosis vaccination did not significantly affect the cecal SCFA composition, therefore, the LefSe analysis was employed to investigate the differences in microbial compositions based solely on dietary treatments. Birds fed the RP-CM and cDDGS diets showed a shared common microbiota comprising four genera − *Alistipes A 871400, Tidjanibacter, QALR01, Massilioclostridium* – along with two families, *CAG-552* and *Acutalibacteraceae*, and an order *Peptostreptococcales* taxon, indicating substantial microbial similarity across these groups. David et al. (2014) reported that the increased abundance of the bile-tolerant genus *Alistipes,* along with elevated fecal concentrations of BCFA, is associated with an animal-based protein diet compared with a plant-based diet. Accordingly, the enrichment of *Alistipes A 871400* observed in birds fed the RP-CM and cDDGS diets may be related to greater amounts of undigested N reaching the hindgut. In contrast, previous studies reported that the enrichment of genera *Faecalicoccus, Turicibacter, Enterocloster,* and family *Ruminococcaceae –* also observed in the RP-cDDGS group of the present study *–* was attributed to the higher dietary fiber contents, hence suggesting a potential role of these microbes in the fiber fermentation (Liu et al., 2021; Petry et al., 2021; Poeker et al., 2018; Sprague et al., 2024). The present findings suggest that the proliferation of these microbes may be influenced by the greater fiber concentrations derived from cDDGS. However, the correlation analysis conducted herein revealed a negative correlation between *Turicibacter* and propionate, a metabolite of fiber fermentation, indicating that these microbes are not necessarily linked to fiber degradation. The significant positive correlation between the genus *Pseudobutyricicoccus* and elevated BCFA concentrations in birds fed the RP-cDDGS diet highlights its potential role as a microbial marker of hindgut protein fermentation in broiler chickens.

## Conclusion

In conclusion, coccidiosis vaccination at placement may impair growth performance through reduced N and AA digestibility. However, beyond the early phase of coccidiosis vaccination, no significant interactions were observed between coccidiosis vaccination and dietary treatment, and vaccination had minimal effects on subsequent growth performance, nutrient digestibility, and cecal metabolite profiles and microbial composition. In contrast, the implementation of RP diets incorporating alternative protein sources such as CM and cDDGS negatively affected ileal nutrient digestibility, increased the flow of indigestible protein to the hindgut, and thereby shifted cecal metabolite and microbial compositions toward increased protein fermentation. These findings highlight that careful formulation of RP diets containing CM and cDDGS is essential to preserve nutrient digestibility and to support a favorable intestinal microbial environment, irrespective of coccidiosis vaccination.

## CRediT authorship contribution statement

**June Hyeok Yoon:** Writing – original draft, Methodology, Data curation. **Adeleye M. Ajao:** Methodology, Investigation. **Shravani Veluri:** Methodology, Investigation. **Revathi Shanmugasundaram:** Writing – review & editing, Resources, Methodology. **Adelumola Oladeinde:** Writing – review & editing, Methodology. **Jeferson Lourenco:** Writing – review & editing, Methodology. **Oluyinka A. Olukosi:** Writing – review & editing, Supervision, Resources, Project administration, Methodology, Investigation, Funding acquisition, Conceptualization.

## Disclosures

The authors declare that they have no known competing financial interests or personal relationships that could have appeared to influence the work reported in this paper.
